# Supplementary material for: Ischemic Etiology and Prognosis in Men and Women with Acute Heart Failure
Source: J Clin Med. 2021 Apr 15;10(8):1713. doi: 10.3390/jcm10081713 (PMC8071524; doi:10.3390/jcm10081713)
Supplement: Supplementary file 1 [file jcm-10-01713-s001.zip › jcm-1171985-supplementary.pdf]

Journal of Clinical Medicine

MDPI

Supplementary materials:

Ischemic etiology and prognosis in men and women with acute heart failure

Lourdes Vicent [1], Jose Guerra [2,3], Rafael Vazquez-García [4], José R. Gonzalez-Juanatey [3,5], Luis Martínez Dolz [6], Javier Segovia [3,7], Domingo Pascual-Figal [8], Ramón Bover [9], Fernando Worner [10], Juan Delgado[1,3], Francisco Fernández-Avilés [3,11,12] and Manuel Martínez-Sellés [3,11,12,13,*]

[1] Cardiology Department, Hospital Universitario 12 de Octubre. Madrid, Spain.
[2] Cardiology Department, Hospital de la Santa Creu i Sant Pau, Barcelona. Spain
[3] CIBER de enfermedades cardiovasculares (CIBERCV), Instituto de Salud Carlos III. Spain.
[4] Cardiology Department, Puerta del Mar University Hospital, Cádiz. Spain
[5] Cardiology Department. University Hospital. Santiago de Compostela. Spain
[6] Cardiology Department. University Hospital La Fe, Valencia. CIBER CV. Spain
[7] Cardiology Department, Hospital Universitario Puerta de Hierro Majadahonda, Madrid, Spain
[8] Cardiology Department, Hospital Virgen de la Arrixaca, Department of Medicine, University of Murcia, Murcia, Spain; 9 Centro Nacional de Investigaciones Cardiovasculares Carlos III (CNIC), Madrid, Spain
[9] Cardiology Department. Hospital Clínico San Carlos. Madrid. Spain
[10] Servicio de Cardiología. Hospital Universitari Arnau de Vilanova. IRBLLEIDA. Lleida. Spain.
[11] Cardiology Department, Instituto de Investigación. Hospital General Universitario Gregorio Marañón. Madrid, España
[12] Universidad Complutense. Madrid, Spain
[13] Universidad Europea. Madrid, Spain
* Correspondence: mmselles@secardiologia.es; Tel.: +34-915-8686-87

Table S1. Basal demographic and clinical characteristics of patients with HF according to sex and etiology.

	**Women with ischemic etiology (N= 167)**	**Women with non-ischemic etiology (N=1018)**	**P**	**Men, ischemic HF (N=446)**	**Men, non-ischemic HF (N= 629)**	**P**
Age (years)	77.2±10.3	74.7±11.7	0.019	71.9±10.4	69.4±13.3	<0.001
Previous heart failure diagnosis	121 (72.5)	327 (55.5)	<0.001	294 (65.9)	318 (50.6)	0.032
Previous heart failure admissions	84 (50.3)	220 (37.4)	0.002	206 (46.2)	235 (37.4)	0.003
Tobacco use						
- *Smoker*	*5 (3.0)*	26 (4.4)	0.660	69 (15.5)	93 (14.9)	0.442
- *Former smoker*	*17 (10.2)*	54 (9.2)	0.685	252 (56.6)	307 (49.1)	0.421
Alcohol consumption	*1 (0.6)*	10 (1.7)	0.241	53 (11.9)	129 (20.5)	0.178
Dyslipidemia	109 (65.3)	303 (51.4)	0.001	320 (71.8)	280 (44.6)	0.042
Diabetes mellitus	*115 (68.9)*	244 (41.4)	<0.001	249 (55.8)	239 (38.1)	<0.001
Hypertension	150 (89.8)	444 (75.4)	<0.001	390 (87.4)	430 (68.5)	<0.001

Chronic kidney disease<br>- GFR <30<br>- GFR 30 - 59	15 (9.0)<br>42 (25.3)	25 (4.3)<br>108 (18.5)	0.010<br>0.014	34 (7.6)<br>129 (28.9)	24 (3.8)<br>123 (19.6)	0.032<br>0.019
Chronic obstructive pulmonary disease	*8 (4.8)*	53 (9.0)	0.150	94 (21.1)	135 (21.5)	0.213
Stroke	17 (10.2)	52 (8.8)	0.785	50 (11.2)	64 (10.2)	0.452
Peripheral arterial disease	22 (13.2)	48 (8.2)	0.117	84 (18.8)	56 (8.9)	0.003
Previous myocardial infarction	117 (70.1)	27 (4.6)	<0.001	295 (66.1)	45 (7.7)	<0.001
Previous coronary artery revascularization<br>Percutaneous<br>Surgical<br>Both	61 (36.5)<br>20 (12.0)<br>9 (5.4)	24 (4.1)<br>15 (2.6)<br>7 (1.2)	<0.001	166 (37.4)<br>63 (14.2)<br>45 (10.1)	31 (5.0)<br>17 (2.7)<br>8 (1.3)	<0.001
Atrial fibrillation	56 (33.5)	288 (28.3)	0.043	145 (32.5)	283 (45.0)	0.021
Previous implantable cardioverter defibrillator	*4 (2.4)*	16 (2.7)	0.881	57 (12.8)	50 (8.0)	0.001
Previous cardiac resynchronization therapy	3 (1.8)	10 (1.7)	0.904	21 (4.7)	17 (2.7)	0.152
Previous treatments:<br>- ACEIs<br>- ARBs<br>- Betablockers<br>- Ivabradine<br>- Loop diuretics<br>- Mineralocorticoid receptor antagonists<br>- Digoxin<br>- Oral anticoagulation	77 (46.1)<br>50 (29.9)<br>118 (70.7)<br>9 (5.4)<br>104 (62.3)<br>38 (22.8)<br>12 (7.2)<br>51 (30.5)	179 (30.4)<br>156 (26.5)<br>282 (48.0)<br>13 (2.2)<br>354 (60.1)<br>124 (21.1)<br>85 (14.4)<br>275 (46.7)	0.001<br>0.223<br><0.001<br>0.132<br>0.337<br>0.136<br>0.032<br>0.001	213 (47.8)<br>103 (23.1)<br>312 (70.1)<br>43 (9.6)<br>287 (64.4)<br>144 (32.3)<br>35 (7.9)<br>162 (36.5)	219 (34.9)<br>135 (21.5)<br>281 (44.6)<br>22 (3.5)<br>333 (53.0)<br>131 (20.8)<br>66 (10.5)<br>232 (37.1)	0.001<br>0.418<br><0.001<br>0.218<br>0.319<br>0.113<br>0.027<br>0.007
Left ventricular ejection fraction (%)	46.6±16.6	53.6±16.0	<0.001	38.6±15.7	42.9±17.6	0.001
Death during hospital admission	8 (4.8)	20 (3.4)	0.227	18 (4.0)	25 (4.0)	0.616
Hospital readmissions due to HF at 12 months	57 (34.1)	181 (30.7)	0.406	146 (32.7)	159 (25.3)	0.009
All-cause mortality at 12 months	42 (25.2)	111 (18.9)	0.081	102 (22.9)	120 (19.1)	0.131
Heart transplant at 12 months	*1 (0.6)*	4 (0.7)	0.528	13 (2.9)	18 (2.9)	0.959
Sudden cardiac death at 12 months	4 (2.4)	10 (1.7)	0.55	18 (4.0)	23 (3.7)	0.750

Death due to refractory heart failure	19 (11.4)	59 (10.0)	0.665	52 (11.7)	44 (7.0)	0.009
Death due to non-cardiac causes	13 (7.8)	25 (4.2)	0.072	24 (5.4)	36 (5.7)	0.893
